# Partial-EMT in oral squamous cell carcinoma: molecular circuitry and clinical translation

**DOI:** 10.1038/s41368-025-00417-0

**Published:** 2026-01-30

**Authors:** Chunhua Wang, Motoharu Sarubo, Siqi Chen, Yasusei Kudo

**Affiliations:** 1 Department of Oral Bioscience, Tokushima University Graduate School of Biomedical Sciences, Tokushima, 770-8504 Japan; 2 Institute of Photonics Human Health Frontier, Tokushima University, Tokushima, 770-8504 Japan

**Keywords:** Cancer microenvironment, Oral cancer, Metastasis

## Abstract

Oral squamous cell carcinoma (OSCC) is a prevalent malignancy with high morbidity and mortality. Globally, about 400 000 people are affected, often with a poor quality of life. Its high mortality is mainly due to its aggressive growth and tendency to spread. Epithelial-mesenchymal transition (EMT) is a central regulatory hub driving tumor cell migration and invasion by enabling changes in cell characteristics. During EMT, epithelial cells gradually take on mesenchymal traits, gaining mobility and spreading more easily. Recent multi-omics studies show that many cancer cells exist in a hybrid or partial-EMT state, which lies between the full epithelial and mesenchymal forms. Cells in this state are especially invasive and metastatic, with high plasticity that promotes tumor progression. This review summarizes the role of partial-EMT in OSCC, with a focus on how it alters the tumor microenvironment (TME), promotes invasion and metastasis, and influences cancer stem cells (CSCs). We also highlight the link between partial-EMT and treatment resistance in OSCC. Based on these insights, we discuss therapeutic strategies targeting partial-EMT to improve outcomes. Targeting partial-EMT may offer promising strategies to enhance treatment effectiveness and improve patient survival and quality of life.

## Background

OSCC is one of the most common malignant tumors worldwide. It disproportionately affects specific subpopulations, particularly in developing countries, where it is a leading cause of death due to oral diseases.^1^ Established risk factors for OSCC include smoking, chewing tobacco, alcohol consumption, and its derivatives, and oral viral infections such as human Papillomavirus (HPV).^2,3^ Despite significant advances in research, the 5-year survival rate for OSCC patients remains below 60%, largely due to its high heterogeneity, aggressive metastatic behavior, and frequent recurrence.^4^ The complex mechanisms underlying OSCC onset and progression, coupled with the absence of precise diagnostic methods and effective therapeutic strategies, contribute to tens of thousands of deaths annually.^5,6^ These challenges highlight the urgent need for the development of innovative diagnostic tools and targeted therapeutic approaches to improve patient outcomes.

**Fig. 1:**
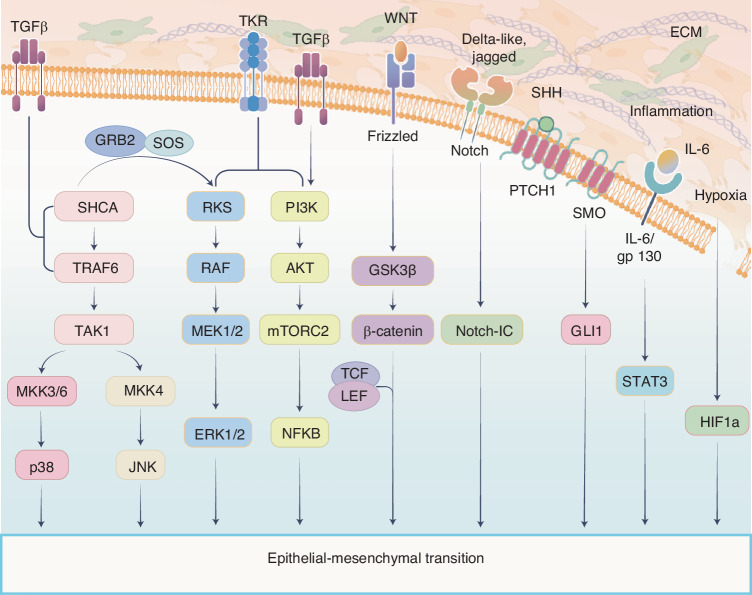
Key Signaling Pathways Promoting the Formation of EMT. Several critical signaling pathways and molecular networks contribute to the initiation and progression of EMT, including integrin, TGF-β, tyrosine kinase receptors (TKRs), WNT, Notch, Sonic hedgehog (SHH), inflammatory factors, and hypoxia signaling. These pathways activate core molecules (such as PI3K, AKT, GSK3β, β-catenin, STAT3, and HIF1α) and their downstream transcription factors (such as TCF/LEF and GLI1), thereby promoting EMT. EMT leads to the loss of cell-cell adhesion, remodeling of the ECM and enhancement of tumor cell migration, driving cancer cell invasion and metastasis. This figure was hand-drawn by the author using Procreate (https://procreate.com)

EMT is a biological process in which epithelial cells lose their polarity and intercellular adhesion, adopting mesenchymal-like properties. EMT occurs naturally during development, wound healing, and inflammation, but it is also a hallmark of cancer progression. In the context of cancer, EMT promotes tumor advancement by conferring a mesenchymal phenotype associated with increased invasiveness and metastatic potential.^7,8^ EMT is not a binary process; instead, cells can occupy an intermediate state between epithelial and mesenchymal characteristics, known as partial-EMT. Cancer cells in this state retain some epithelial traits while acquiring mesenchymal-like properties, enabling them to behave similarly to mesenchymal cells without fully transitioning (Fig. 1).^9^ With advancements in sequencing technologies, it has become evident that cancer cells in the partial-EMT state exhibit enhanced invasiveness and metastatic potential.^10^ Studies have shown that partial-EMT plays a pivotal role in tumor tissue invasion, collective migration, the formation of circulating tumor cells (CTCs), and metastasis.^11^ Clinically, these characteristics are significant as they contribute to multi-drug resistance in tumor cells, adversely affecting patient survival and quality of life.^12^ The partial-EMT state is regulated by intrinsic factors, including genetic, epigenetic, and post-translational modifications of tumor cells, as well as extrinsic factors such as paracrine signaling from stromal cells within the TME (Fig. 1).^13,14^ A deeper understanding of partial-EMT is critical to elucidate how epithelial plasticity drives tumor invasion, immune exclusion, and therapy resistance. Although meaningful advances have been made, key mechanistic and translational gaps remain and warrant continued investigation.

In this review, we highlight the role of partial-EMT in tumor progression and metastasis of OSCC, with a particular focus on its interactions with the TME. Additionally, we summarize the crosstalk between partial-EMT and key aspects of OSCC biology, including metastasis, CSCs, heterogeneity, and drug resistance. Finally, we provide a comprehensive overview of potential therapeutic and diagnostic biomarkers for OSCC, emphasizing strategies targeting partial-EMT. Future research into partial-EMT remains essential, as targeting this critical process holds promise for improving treatment outcomes and significantly enhancing the quality of life for cancer patients.

## EMT and partial-EMT in OSCC

Canonical EMT in OSCC is characterized by near-complete loss of membranous E-cadherin, dissolution of apical–basal polarity, robust induction of Vimentin (VIM) and N-cadherin, and sustained activation of EMT-inducing transcription factors such as SNAIL, TWIST, and Zinc finger E-box binding homeobox (ZEB). These cells acquire a mesenchymal morphology, migrate as dispersed single cells, and typically show reduced proliferative indices—features linked to dissemination and therapy resistance.^13^ By contrast, partial-EMT represents an intermediate state in which epithelial lineage identity and cytokeratin expression (e.g., Keratin 8/14: KRT8/14) are preserved, while motility, matrix-interaction, and stress-response programs are activated. Membranous E-cadherin is attenuated but not lost,^15^ and mesenchymal markers (VIM and N-cadherin) appear in subsets of cells. Matrix-remodeling and stromal-interaction genes such as laminin γ2 (LAMC2), Integrin alpha-5 (ITGA5), podoplanin (PDPN), Matrix metalloproteinases (MMPs), Lysyl oxidase (LOX), fibroblast activation protein (FAP), and platelet-derived growth factor receptor beta (PDGFRB) are upregulated.^16^

In OSCC, partial-EMT arises from the convergence of soluble, mechanical, and metabolic cues onto a restricted transcriptional–epigenetic core. Canonical Transforming growth factor-β (TGF-β) signaling activates SMAD2/3–SMAD4 to induce SNAIL, snail family transcriptional repressor 2 (SNAI2), and ZEB1/2 while repressing epithelial gatekeepers (Grainyhead-like 2: GRHL2 and Ovo-like transcriptional repressor 1: OVOL1).^17^ Non-canonical TGF-β pathways engage Phosphatidylinositol 3-kinase (PI3K)/AKT and Mitogen-activated protein kinase (MAPK) cascades and cooperate with Wnt/β-catenin and Hippo YAP/TAZ signaling, converging on modules centered on SNAI2, ZEB, TWIST, and Activator protein 1 (AP-1).^18^ At collagen-dense invasive fronts, integrin–Focal adhesion kinase (FAK) mechanotransduction drives nuclear YAP/TAZ activity, while hypoxia-inducible factor 1 alpha (HIF-1α) further upregulates SNAIL and TWIST and reshapes cytokine networks.^19–21^ Together, these signals stabilize the hybrid state, marked by genes such as LAMC2, ITGA5, PDPN, LOX, and TGF-β induced (TGFBI). Single-cell and histological studies reveal that partial-EMT is enriched at invasive fronts of head and neck squamous cell carcinoma (HNSCC), characterized by markers such as laminin β3 (LAMB3), MMP10, and TGFBI. This program correlates with infiltrative growth, nodal metastasis, and poor prognosis, and functionally supports collective invasion with a leader–follower organization.

The two states differ in spatial ecology. EMT tends to arise in scattered foci, whereas partial-EMT consistently localizes to collagen-rich invasive margins where tumor cells interact with myofibroblastic and inflammatory cancer-associated fibroblast (CAF) niches and Secreted phosphoprotein 1 (SPP1)-positive macrophages. This front-dominated distribution contributes to immune exclusion at the formation of collectively migrating clusters capable of seeding nodal metastases. Because of this localization, partial-EMT may be underdetected by bulk assays, requiring multiplex Immunohistochemistry (IHC)/Immunofluorescence or spatial transcriptomic approaches anchored to tumor margins.^22^

The distinction has both diagnostic and therapeutic implications. EMT is typically characterized by the loss of membranous E-cadherin and diffuse expression of VIM, whereas partial-EMT is best recognized by the co-expression of epithelial markers (such as epithelial cell adhesion molecule [EpCAM], E-cadherin, and cytokeratins [CKs]) and mesenchymal or matrix-interaction markers (including VIM, N-cadherin, LAMC2, ITGA5, PDPN, MMPs, LOX, FAP, and PDGFRB), often localized at the leading-edge of tumors. Histologically, it corresponds to partial E-cadherin loss, preserved cell–cell contacts, and a collective-migration phenotype. Transcriptomic profiling typically places these cells in the intermediate EMT range or enriches for the head and neck partial-EMT program.^23^ Early partial-EMT reflects initial E-cadherin attenuation with nascent VIM/N-cadherin, while late partial-EMT shows broader induction of mesenchymal and matrix-interaction programs.^24^ Therapeutically, targeting TGF-β or matrix-sensing pathways may be most effective at partial-EMT-rich margins. Trial designs should incorporate spatially informed enrichment and longitudinal pharmacodynamic monitoring to avoid misclassification and capture compensatory signaling circuits. Clarifying these distinctions emphasizes the clinical importance of partial-EMT in OSCC and aligns biomarker development with biology at the invasive front.

## Crosstalk between partial-EMT and TME

The TME is composed of cellular and noncellular components that provide a supportive niche for tumor survival, proliferation, and metastasis. Key elements of the TME include immune cells, fibroblasts, blood vessels, cytokines, and extracellular matrix (ECM) components.^15,16^^,25,26^ This highly dynamic structure constantly adapts to the evolving needs of the tumor or environmental cues, influencing tumor progression. To acquire invasive capabilities, tumor cells often undergo partial-EMT or full EMT at specific tissue sites. Among these processes, the induction of partial-EMT is particularly influenced by the TME.^17^^,27^ The intricate interactions between tumor cells and the TME play a pivotal role in driving partial-EMT, facilitating tumor invasion, and shaping the metastatic cascade.

## Partial-EMT and immunosuppressive cells

The TME can be categorized into immune-suppressive, inflammatory, hypoxic, and stromal microenvironments, all of which contribute to tumor progression. Among these, the immune-suppressive TME plays a particularly important role in promoting partial-EMT. This environment inhibits normal immune cell functions while supporting the proliferation and activity of pro-tumor immune cells.

The tumor-associated macrophages (TAMs) are the key immune-suppressive components of the TME. In OSCC, major TAM subsets include M1-like, M2-like, and SPP1⁺ populations.^28,29^ Bednarczyk et al. reported that secretory factors from M1-like TAMs induce partial-EMT in breast cancer cells, enhancing their proliferation and invasiveness.^30^ In M2-like TAMs, lactic acid promotes polarization and induces the secretion of glycoprotein non-metastatic protein B (GPNMB), which binds to CD44 on OSCC cells and activates the PI3K/AKT/mechanistic target of rapamycin (mTOR) signaling pathway, thereby promoting partial-EMT.^31^ Cao et al. further demonstrated that Tweety family member 3 (TTYH3) is upregulated in OSCC cells, enhancing tumor–macrophage crosstalk and M2 polarization. Knockdown of TTYH3 suppressed partial-EMT, suggesting its pivotal role in this process.^32^ SPP1⁺ TAMs represent another critical subset. Partial-EMT tumor cells co-localize with SPP1⁺ macrophages at the invasive front, where SPP1–CD44 and Collagen type I alpha 1 (COL1A1)–CD44 interactions form a pro-invasive niche that stabilizes the partial-EMT state.^33^ Li et al. found that enrichment of SPP1⁺ TAMs correlates with partial-EMT dominance and reduced immune infiltration. These TAMs also cooperate with cancer-associated myofibroblasts (myCAFs) to drive ECM deposition and EMT, creating a stiff and immunosuppressive stroma.^34^ Mechanistically, TAM-derived SPP1 engages CD44 and integrin receptors on tumor or stromal cells and works in concert with myCAF-mediated mechanical cues and TGF-β signaling to reinforce mesenchymal programs.^35^ Under hypoxic and acidic conditions, HIF-1α/Signal transducer and activator of transcription 3 (STAT3) upregulates SPP1 in TAMs, which binds integrin αvβ3 on tumor cells, promoting partial-EMT and metastasis. In addition to TAMs, myeloid-derived suppressor cells (MDSCs) are highly enriched in patients with metastasis. The CD14^+^CD15^+^ monocytic subset of MDSCs is significantly associated with partial-EMT through the induction of reactive oxygen species, which may act as key drivers of this process.^36^

**Fig. 2:**
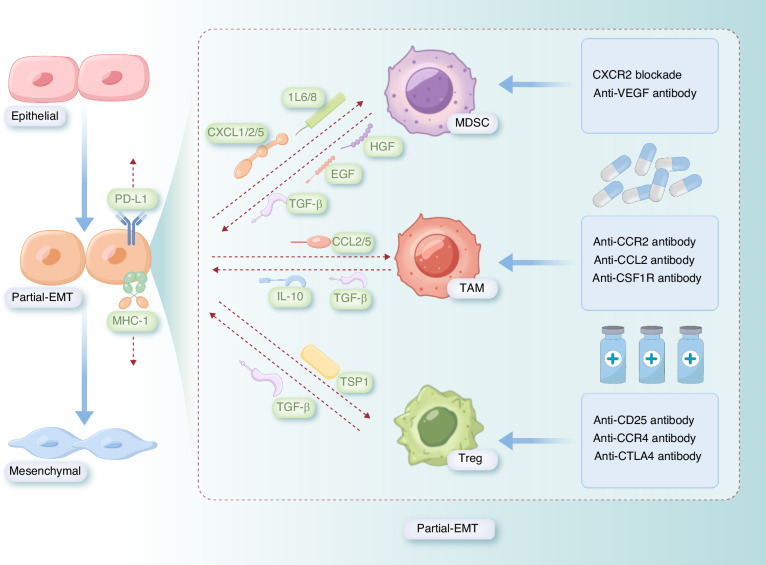
Formation of an Immune-Suppressive Microenvironment Mediated by partial-EMT and Targeting Immune-Suppressive Cells. During partial-EMT, tumor cells induce immune evasion, promoting tumor progression. At the Partial-EMT stage, cells evade immune surveillance by upregulating PD-L1 and downregulating MHC-I, thereby enhancing tumor immune evasion. Additionally, partial-EMT-related signals (including CXCL1/2/5, IL-6/8, EGF, TGF-β, and CCL2/5) recruit and activate immunosuppressive cells, such as MDSCs, TAMs, and Tregs. Targeted therapeutic strategies aimed at these immunosuppressive cells include CXCR2 inhibitors, anti-VEGF antibodies, anti-CCR2 antibodies, anti-CCL2 antibodies, anti-CSF1R antibodies, anti-CD25 antibodies, anti-CCR4 antibodies, and anti-CTLA4 antibodies. These strategies represent potential therapeutic approaches to overcome partial-EMT-mediated immunosuppression. This figure was hand-drawn by the author using Procreate (https://procreate.com)

During partial-EMT, tumor cells evade immune surveillance by upregulating immune checkpoint molecules, such as PD-L1 and downregulating antigen-presenting molecules like MHC-I. This enhances immune evasion and tumor progression. Furthermore, partial-EMT-associated cytokines and chemokines—including C-X-C motif chemokine ligand 1/2/5 (CXCL1/2/5), Interleukin-6/8 (IL-6/8), Epidermal growth factor (EGF), TGF-β, and C-C motif chemokine ligand 2/5 (CCL2/5)—facilitate the recruitment and activation of immunosuppressive cells such as MDSCs, TAMs, and regulatory T cells (Tregs), thereby reinforcing the immune-suppressive microenvironment (Fig. 2).

In turn, partial-EMT itself contributes to the establishment of this immunosuppressive niche (Fig. 2), enabling tumor cells to further escape immune detection and drive metastasis. Given this bidirectional relationship, targeting immunosuppressive cells has emerged as a promising strategy to counteract partial-EMT-driven immune evasion. Potential therapeutic interventions include inhibitors or antibodies targeting C-X-C motif chemokine receptor 2 (CXCR2), Vascular endothelial growth factor (VEGF), C-C chemokine receptor type 2 (CCR2), CCL2, Colony stimulating factor 1 receptor (CSF1R), CD25, CCR4, and Cytotoxic T-lymphocyte antigen-4 (CTLA4), which may disrupt the partial-EMT–immunosuppression axis and enhance antitumor immunity (Fig. 2).

## Partial-EMT and the hypoxic microenvironment

Hypoxia within tumor tissues is a key driver of partial-EMT. It promotes this hybrid phenotype by regulating critical molecular pathways. For instance, hypoxia-responsive genes, such as eukaryotic translation initiation factor 5A2 (EIF5A2) upregulate HIF1α and mesenchymal markers like VIM in esophageal squamous cell carcinoma (ESCC).^37^ The partial-EMT phenotype in ESCC is characterized by the co-expression of mesenchymal markers (e.r., VIM) and VEGF, with HIF1α acting a central regulatory role.^38^ Singh et al. reported that metastatic tumors under hypoxic conditions exhibit elevated levels of HIF1α, VIM, and fibronectin 1 (FN1), along with an unexpected upregulation of E-cadherin, further supporting the presence of partial-EMT.^39,40^ Their study also noted that tumor cells in hypoxic regions were predominantly in a state of abnormal proliferation, with only a subset in the S phase of the cell cycle. Partial-EMT cells were mainly localized at the tumor periphery, suggesting that hypoxia may facilitate collective migration, thereby promoting tumor invasion and metastasis.

The interplay between hypoxia and non-coding RNAs (ncRNAs) further sustains and stabilizes partial-EMT, particularly at the invasive front. In pancreatic ductal adenocarcinoma (PDAC), hypoxia strongly induces the long non-coding RNA (lncRNA) MIR31HG, which promotes features of the basal-like subtype and supports the partial-EMT state, stabilizing the hybrid epithelial-mesenchymal phenotype.^41^ A similar hypoxia–ncRNA interaction is likely operative at the invasive front in OSCC. In an esophageal cancer model, MIR503HG relieves repression of Fos-like antigen 1 (FOSL1), promoting EMT traits, ECM deposition, and autophagy—mechanisms that support matrix adaptation and stress tolerance in partial-EMT-rich regions. In OSCC, circFNDC3B (Fibronectin type III domain containing 3B) is upregulated and associated with nodal metastasis.^42^ It enhances angiogenesis via the Focused ultrasound (FUS)–MDM2–HIF1A–VEGFA pathway, promotes lymphangiogenesis through the miR-181c-5p/PROX1 axis, and increases Serpin family E member 1 (SERPINE1) expression by sponging microRNAs, collectively reinforcing partial-EMT features. These findings illustrate how the hypoxic TME amplifies and sustains the partial-EMT phenotype, facilitating tumor dissemination through vascular, lymphatic, and matrix routes.

## Partial-EMT and ECM remodeling

The ECM plays a pivotal role in regulating the partial-EMT phenotype. Composed of structural components such as collagen, non-collagenous proteins, and elastin, the ECM influences partial-EMT by modulating cell adhesion, migration, and intracellular signaling pathways. These dynamic interactions not only drive tumor invasion and metastasis but also reinforce the stability of the partial-EMT state.^43^

In OSCC, Sun et al. demonstrated that the transcription factor BARX Homeobox 2 (BARX2), which is essential for actin dynamics, cell adhesion, and cytoskeletal remodeling, is significantly downregulated. Reduced BARX2 expression enhances SERPINE2 activity, which correlates with increased invasiveness and partial-EMT. Mechanistically, BARX2 normally upregulates miR-186-5p and miR-378a-3p, both of which suppress SERPINE2. Loss of this regulation predisposes OSCC cells to acquire partial-EMT traits.^44^ In hepatocellular carcinoma cells, SNAI2 overexpression has been linked to exosome-derived ECM components, including FN1 and collagen type II alpha 1 (COL2A1), both of which support the maintenance of the partial-EMT state.^45^ Likewise, laminin 5 (LN5) has been shown to independently induce partial-EMT and act synergistically with transforming growth factor-beta 1 (TGFB1) to stabilize the phenotype.^46^

ECM remodeling also significantly impacts the migratory behavior of partial-EMT cells. In pancreatic cancer, dynamic ECM rearrangement mediated by integrin β1 and FAK, is essential for partial-EMT induction.^47^ In colorectal cancer (CRC), low expression of Neogenin1 alters the expression of ECM-related proteins such as ITGA2, ITGA5, and FN1, further promoting partial-EMT.^48^ Partial-EMT cells often migrate collectively and are closely associated with ECM remodeling. In 3D culture models of salivary gland adenoid cystic carcinoma, tumor spheroids upregulate cathepsin B (CTSB), RhoA, and FAK, thereby modifying the ECM to create a mechanically permissive environment for migration.^49^ Together, these findings underscore the dual role of ECM remodeling—providing both biochemical signals and mechanical cues—in driving partial-EMT, enhancing tumor invasiveness, and facilitating the adaptation of tumors cells to their microenvironment.

## Partial-EMT and CAFs

CAFs are pivotal components of the TME, playing a critical role in tumor progression, metastasis, and ECM remodeling. CAFs promote the transition of tumor cells into a partial-EMT state through various molecular pathways and reciprocal interactions, thereby enhancing tumor aggressiveness. These interactions foster a pro-tumorigenic microenvironment. In regions like Asia, where betel nut chewing is common, oral submucous fibrosis (OSF) increases the risk of OSCC. During the progression from OSF to OSCC, metabolic reprogramming and partial-EMT contribute to a microenvironment rich in CAFs and collagen deposition. These components interact with immune and tumor cells, suppressing normal immune response and facilitating tumor invasion and metastasis.^50^ CAFs are not a homogeneous population but consist of plastic, interconvertible lineages. The prevailing classification divides them into three major subtypes: i) myCAFs: Characterized by high expression of α-SMA and transgelin (TAGLN),^51^ ii) inflammatory CAFs (iCAFs): Upregulate IL-6, Leukemia inhibitory factor (LIF), and CXCL12, express low α-SMA, and are typically located farther from tumor nests,^52^ and iii) antigen-presenting CAFs (apCAFs): Express MHC class II and antigen-processing genes, but provide insufficient co-stimulation, which predisposes local CD4⁺ T cells to hyporesponsiveness.^53,54^

Shan et al. demonstrated that CAFs promote partial-EMT by upregulating the Hedgehog signaling pathway, which subsequently suppresses E-cadherin expression.^55^ Reciprocal interactions between CAFs and partial-EMT tumor cells drive OSCC progression. Single-cell RNA sequencing (scRNA-seq) of OSCC revealed strong intercellular communication between two cell populations. Tumor necrosis factor superfamily member 12 (TNFSF12), expressed on myCAFs, interacts with TNFRSF25 and TNFRSF12A on partial-EMT tumor cells, enriching metastasis-associated signaling pathways.^56^ This suggests that CAFs directly enhance the invasive and metastatic potential of partial-EMT tumor cells. Evidence also shows that lower Wnt activity promotes the induction of iCAFs, which, in organoid co-culture, upregulate partial-EMT markers in cancer cells. In contrast, higher Wnt activity favors a myCAF phenotype and can reverse partial-EMT features induced under low-Wnt conditions.^57^ Liu et al. reported that the stroma associated with partial-EMT tumors was enriched for microvessel density, iCAFs, and hyaluronan deposition, highlighting the decisive role of matrix remodeling at the invasive front in collective migration.^58^ They identified two stroma-derived biomarkers: TGFBI, associated with iCAF, which predicted poorer survival, and hyaluronidase 1 (HYAL1), associated with endothelial cells, which predicted better survival. Both biomarkers were validated across pan-cancer datasets. In HNSCC, a study delineated two fibroblast subsets: LOX⁺ iCAFs and LOX^−^ myCAFs.^59^ The LOX⁺ iCAF signature correlated with activation of partial-EMT pathways, an inflamed, immunosuppressive TME, and poorer prognosis, suggesting its potential as a predictor of immunotherapy response. Silencing LOX in CAFs reduced tumor-cell proliferation and migration and suppressed partial-EMT by attenuating the IL-34/Colony-stimulating factor 1 receptor (CSF1R)/AKT signaling pathway. Punovuori et al. quantified tumor phenotypic diversity in detail using multiparametric imaging combined with cell state markers at single-cell resolution.^60^ They also uncovered interaction patterns between tumor cells and mesenchymal stromal cells through spatial transcriptomics and tumor organoid dynamics. Notably, tumor cells in the partial-EMT state exhibited heightened sensitivity to mesenchymal components within the TME. Interaction with CAFs induced the transition of partial-EMT tumor cells to a more aggressive phenotype, marked by EGF receptor (EGFR) and its ligand amphiregulin (AREG), mediating dynamic crosstalk with CAFs to further enhance tumor aggressiveness. The presence of partial-EMT tumor cells correlated with CAF abundance and poorer clinical outcomes in HNSCC.^60^

In CRC cell lines, CAFs promote collagen production and ECM remodeling, while enhancing glycolysis, thereby contributing to a hypoxic TME. ScRNA-seq revealed that CAFs with high expression of ECM components, ECM-remodeling enzymes, glycolysis-related genes, and immunosuppressive factors, induce partial-EMT in a subpopulation of CRC cells.^61^

## Role of partial-EMT in tumor collective migration and metastasis

Tumor cells in the partial-EMT state acquire mesenchymal traits while retaining epithelial markers and functions.^22^ This hybrid phenotype enables them to maintain certain cell–cell junctions, such as tight and adherens junctions, which facilitates collective migration.^62,63^ Hsiao et al. demonstrated that in OSCC, miR-455-5 induces partial-EMT by suppressing PDZ domain containing 1 interacting protein 1 (PDZK1IP1) and upregulating VIM, promoting collective tumor cell migration.^64^ Key transcription factors also regulate partial-EMT induction.^65^ For example, Snail promotes collective migration by upregulating claudin-11 (CLDN11), which activates Src, and induces expression of Cysteine-rich angiogenic inducer 61 (Cyr61), increasing RhoA activity while maintaining cell adhesion.^66^

TGF-β plays a crucial role in fine-tuning epithelial–mesenchymal plasticity, rather than enforcing a binary switch. In breast cancer models, TGF-β1 induces a hybrid program characterized by increased CLDN1, which preserves junctional integrity within migrating clusters and enables collective migration.^67^ Simultaneously, TGF-β1 downregulates SMAD1/5, repressing Bone morphogenetic protein-2 (BMP-2) signaling that would otherwise favor Mesenchymal-epithelial transition (MET) by curbing N-cadherin and Snail. This shift promotes partial-EMT.^68^ Additionally, reduced SMAD3 activity combined with MYC activation also facilitates collective migration under TGF-β signaling.^69^ These findings identify TGF-β as a key driver of partial-EMT–dependent collective invasion at the invasive front.^70^

Partial-EMT cells exhibit dynamic adaptability to the TME by regulating cell–cell junctions and undergoing cytoskeletal reorganization. However, understanding the molecular mechanisms underlying their collective migration requires further investigation using integrated multi-omics and advanced sequencing technologies.^71^

During OSCC metastasis to bone, the transcription factor Twist promotes tumor infiltration by inducing partial-EMT.^72^ Similarly, in laryngeal squamous cell carcinoma, forkhead box D1 (FOXD1) facilitates metastasis to proximal lymph nodes by upregulating zinc finger protein 532 (ZNF532), which drives partial-EMT and enhances metastatic potential.^73^

Tumor budding refers to single tumor cells or clusters (up to four cells) at the invasive front. It corresponds to a hybrid phenotype that retains epithelial lineage identity while upregulating motility and matrix-interaction programs, making it closely linked to partial-EMT.^74^ In breast cancer, NF-E2-related factor 2 (NRF2) upregulation prevents completion of the EMT cascade, stabilizes cells in a partial-EMT state, and correlates with poorer survival. In urothelial carcinoma, sequential IHC combined with digital image analysis detects partial-EMT cells at diagnosis, predicting prognosis. In PDAC, IHC with budding assessment shows induction of partial-EMT at the tumor–stroma interface and within buds. In CRC, budding and poorly differentiated clusters represent distinct morphological manifestations along a partial-EMT continuum.^75^

In OSCC, laser capture-based profiling shows that budding cells are enriched for TGF-β–mediated EMT programs, upregulating ZEB1, Paired related homeobox 1 (PRRX1) and downregulating OVOL1 and the miR-200 family, consistent with epithelial plasticity.^76^ Patient-derived metachronous OSCC cell lines show that TGF-β signaling at the deep invasive front induces LAMC2, enhancing migration and invasion, with the high-budding line exhibiting the strongest response. Partial weakening of junctional adhesion and induction of EMT transcription factors provide an entry point for collective migration. In human pluripotent stem cells, CRISPRi-mediated reduction or delocalization of E-cadherin triggers partial-EMT: cells retain epithelial identity while upregulating SNAI1/SNAI2, β-catenin relocates to the cytoplasm and nucleus, and collective migratory capacity increases. This provides a causal reference for group invasion at OSCC margins.^77^ In OSCC, miR-455-5p downregulates PDZK1IP1, amplifies TGF-β signaling, and increases VIM, enhancing migration and collective movement.^64^ These changes align with higher partial-EMT scores and greater risks of nodal metastasis and recurrence. In HNSCC, budding cells are governed by a Cytoskeleton regulator RNA (CYTOR)–FOSL1 axis, where CYTOR promotes FOSL1 condensates and activates pro-metastatic gene programs, linking partial-EMT to early dissemination.^78^

Comparisons of microvascular density between budding and non-budding regions, or between high- and low-budding tumors, do not consistently reveal a significantly increase. This suggests that budding reflects a cell-state transition supported by a stromal niche rather than a purely angiogenic phenomenon.^79^ Histologically, high budding correlates with an infiltrative invasion, greater depth of infiltration, increased risk of nodal metastasis, and poorer survival, underscoring its value as an independent prognostic marker. Tumor budding thus serves as a morphologic readout of partial-EMT at the invasive front, converging with TGF-β–driven transcriptional programs, matrix remodeling, and CAF-enriched stromal niches. Methodologically, standardized Hematoxylin-Eosin (HE) staining-based bud counting at annotated margins should be paired with multiplex IHC or spatial transcriptomics to quantify partial-EMT and matrix programs.^80^ Practical panels include co-expression of EpCAM and E-cadherin with VIM or N-cadherin, alongside features like LAMC2, ITGA5, PDPN, MMPs, and LOX. Clinically, integrating bud density with partial-EMT molecular readouts into a margin-anchored composite score enables risk stratification, longitudinal monitoring, and patient enrichment in trials targeting TGF-β or matrix-sensing pathways in combination with immunotherapy.^81,82^ Thus, budding emerges not as an isolated histopathological finding but as an actionable indicator aligned with partial-EMT biology, clinical prognosis, and therapeutic translation.

## Partial-EMT and tumor cell stemness

In the partial-EMT state, tumor cells acquire stem cell-like properties, including self-renewal and differentiation potential. These traits enhance cellular adaptability, invasiveness, and resistance to therapy. Partial-EMT cells exhibit increased invasiveness and contribute to metastasis while acquiring CSC features.^83,84^ CSCs are a subpopulation with self-renewal and differentiation capacity, driving tumor heterogeneity and resistance to treatment.^51,85^ At metastatic sites, CSCs retain tumor-initiating potential and exhibit heightened resistance to cell death and therapy.^77^

Research indicates that transcription factors such as Snail and Twist1 promote stemness in breast cancer cells by upregulating stem cell markers like CD44 and CD24, which are closely associated with tumorigenic potential.^85,86^ Similarly, Snail and ZEB1/2 enhance stem-like properties by remodeling the cytoskeleton and disrupting cell–cell junctions. In SCC, aberrant HIPPO signaling drives the expression of CD44 and SRY-related HMG-box 2 (SOX2), which are critical for maintaining self-renewal and stemness in tumor cells.^87^ Additionally, CD44 facilitates ECM adhesion, supporting tumor cell survival across diverse TME.

TGF-β signaling is another key pathway linking partial-EMT to CSC programs. In HNSCC, TGFBI upregulates TAGLN, reinforcing the hybrid phenotype while promoting CSC-like traits.^88^ In non-small cell lung cancer (NSCLC), TGF-β1 induces CD133, driving the conversion of non-stem cells into CSCs. CD133 expression is further associated with enhanced invasiveness and drug resistance.^89,90^ These findings suggest that TGF-β integrates adhesion and cytoskeletal remodeling with self-renewal pathways, making it a central regulator of both partial-EMT and CSC properties.^88^

In various cancers, activation of the Wnt and EGFR pathways upregulates transcription factors like OvoL family proteins (e.g., Shavenbaby, Svb), which promote CSC proliferation, differentiation, and migration.^91^ Additionally, aldehyde dehydrogenase (ALDH), a stemness marker, is enriched in partial-EMT breast cancer cells. ALDH^+^ populations display enhanced plasticity, self-renewal, and the potential to form malignant subclones.^92^ By promoting tumor cell self-renewal, modulating the TME, and activating stemness-associated pathways, partial-EMT endows tumor cells with enhanced stem-like properties across multiple cancer types. This contributes to metastasis, therapeutic resistance, and disease recurrence, underscoring the importance of targeting partial-EMT and stemness pathways for more effective cancer treatment strategies.

## Partial-EMT and treatment resistance in cancer therapy

Treatment resistance remains a significant challenge in clinical oncology. Despite advancements in chemotherapy and immunotherapy, tumor cells undergoing partial-EMT exhibit stem cell-like properties that enhance their plasticity in both proliferation and differentiation. This plasticity contributes to resistance against conventional therapies, rendering treatments such as chemotherapy and immunotherapy largely ineffective, particularly for tumor cells with strong partial-EMT-associated stemness.^93^

Partial-EMT cells are also characterized by high expression of the immunosuppressive molecule programmed cell death 1-ligand 1 (PD-L1), which inhibits T-cell-mediated immune response.^94^ Circulating tumor cells that exhibit both PD-L1 positivity and a partial-EMT phenotype are highly resistant to drugs, significantly reducing the efficacy of treatment.^95^

Biddle et al. demonstrated that both epithelial and mesenchymal tumor cell subpopulations are sensitive to chemotherapy agents such as cisplatin in OSCC. However, the partial-EMT subpopulation, marked by high CD44 expression and low levels of EpCAM and CD24, exhibits strong resistance to cisplatin.^96^ Similarly, aberrant activation of the Wnt/β-catenin pathway upregulates EpCAM expression, driving tumor cells into the partial-EMT state in breast cancer.^97^ This transition enhances the secretion of breast cancer resistance proteins, which promote resistance to mitoxantrone.^98^

Furthermore, the upregulation of partial-EMT-related proteins, such as Slug and VIM, has been linked to increased cisplatin resistance. Interestingly, overexpression of Krüppel-like factor 4 (KLF4) reduces Slug levels and enhances tumor cell sensitivity to cisplatin, suggesting that KLF4 could potentially reverse cisplatin resistance by inhibiting the partial-EMT pathway.^99^ In prostate cancer, docetaxel is a primary therapeutic option, but resistance often develops in conjunction with partial-EMT. This resistance is associated with high expression of MMP-1 and ZEB1/2, which enhance CSC properties and tumor invasiveness. ZEB1, a key regulator of partial-EMT, promotes resistance by inhibiting E-cadherin expression. Its levels are significantly elevated in tumor samples after docetaxel treatment.^100^ Targeting partial-EMT-specific transcription factors or signaling pathways may reduce resistance, thereby preventing recurrence and metastasis.^101^ The link between partial-EMT and CSCs also presents opportunities for novel therapeutic approaches, such as targeting stemness-regulating molecules or enhancing immune system function. Mechanisms such as tumor cell stemness, immune evasion, and collective migration enable partial-EMT cells to adapt and survive, making them particularly difficult to eradicate.^93^

In summary, partial-EMT plays a central role in treatment resistance by enhancing tumor cell survival and adaptability. A deeper understanding of the molecular mechanisms driving partial-EMT could pave the way for more effective therapeutic strategies, overcoming drug resistance and ultimately improving the efficacy of cancer treatments.

## Heterogeneity and plasticity of partial-EMT

Partial-EMT represents a dynamic, intermediate state between epithelial and mesenchymal phenotypes. Cancer cells in this state exhibit a combination of characteristics that allow them to diverse microenvironments, contributing to intratumoral heterogeneity, and resistance to treatments. The activation of partial-EMT is shaped by spatiotemporal niches and patient-specific regulatory networks. In early-stage NSCLC, lymph-node-positive tumors are enriched for partial-EMT programs. The hypoxia marker Carbonic anhydrase IX (CAIX) emerges as an independent prognostic factor, highlighting the selective amplification of the hypoxia–TGF–transcription factor module during early dissemination.^102^

**Fig. 3:**
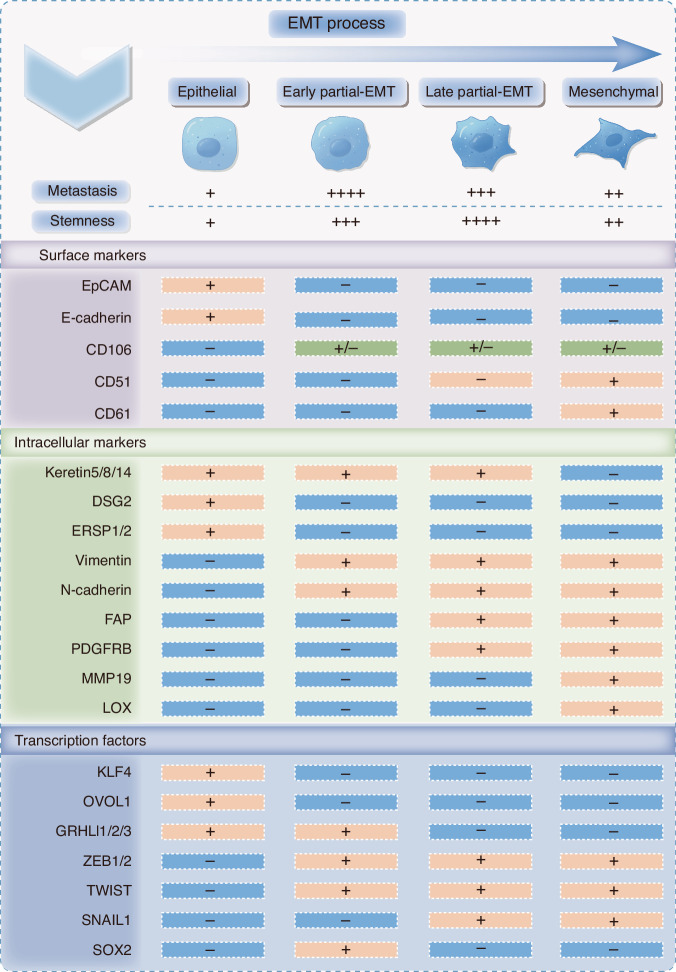
Characteristics of each state and biomarkers during EMT progression. EMT is not binary process but rather a gradual program. The intermediate stage of EMT is referred to as partial-EMT. Cancer cells in this state exhibit both epithelial and mesenchymal features, contributing to tumor progression through metastatic potential and stemness. The epithelial, early partial-EMT, late partial-EMT, and Mesenchymal states are characterized by distinct surface markers, intracellular markers, and transcription factors. This figure was hand-drawn by the author using Procreate (https://procreate.com)

Partial-EMT cells retain a blend of epithelial and mesenchymal traits, expressing epithelial markers in certain contexts while acquiring mesenchymal features, such as enhanced motility and invasiveness. This duality facilitates the formation of heterogeneous tumor subpopulations with increased adaptability and invasiveness. Blanpain et al. provided foundational insights into the EMT spectrum, identifying tumor cells in both early and late partial-EMT states. Early partial-EMT cells exhibit markers like CD106, with low expression of CD51 and CD61, while late-stage cells show high levels of CD106 and CD51 with low CD61. This progression correlates with increased metastatic potential and cellular heterogeneity. Tumor cells in the late partial-EMT state exhibit greater stemness and drug resistance compared to fully epithelial or mesenchymal cells (Fig. 3).^103^ Puram et al., highlighted the clinical significance of partial-EMT in HNSCC, showing that high levels of partial-EMT strongly correlate with lymph node metastasis, lymphovascular invasion, and poor patient outcomes. This underscores the key role of partial-EMT in promoting tumor progression.^104^

Simeonov et al. utilized CRISPR-Cas9-based lineage-tracking tools, such as macsGESTALT, to explore EMT in lung and pancreatic cancers.^105^ They identified distinct intermediate states within partial-EMT, linking the expression profiles of these cells to patient prognosis. Late partial-EMT states were strongly associated with enhanced metastatic properties and poor patient outcomes. Yang et al., expanded on the concept of epithelial-mesenchymal plasticity, revealing that tumor cells can simultaneously display key epithelial and mesenchymal markers.^106^ In early SCC, cells co-expressed CK14 and VIM, with some still retaining E-cadherin expression despite undergoing EMT. This challenges traditional views, which emphasize E-cadherin loss as a hallmark of EMT, highlighting the nuanced continuum of epithelial and mesenchymal traits in partial-EMT.

The heterogeneity and plasticity of partial-EMT cells enhance their ability to adapt to diverse microenvironments, driving metastasis and resistance to treatment. A deeper understanding of the mechanisms and markers associated with partial-EMT states could enable the development of targeted therapies to counteract its role in cancer progression and ultimately improve patient outcomes.

## Biomarkers of partial-EMT: insights and prognostic implications

The exploration of partial-EMT biomarkers is crucial for understanding the mechanisms underlying this intermediate state and assessing patient prognosis. Significant research efforts are being dedicated to this area, revealing insights into the molecular features of partial-EMT. In studies of OSCC, partial-EMT tumor cells identified through IHC exhibited co-expression of E-cadherin and VIM.^107^ In a cohort of nearly 200 patients, approximately 43.5% of clinical samples displayed partial-EMT features.^108^ Similarly, in the HSC-3 OSCC cell line, partial-EMT cells showed elevated N-cadherin expression without a complete loss of E-cadherin. Interestingly, typical EMT-related transcription factors were not significantly upregulated in these cells, suggesting a distinct regulatory mechanism in partial-EMT.^109^

In SCC, tumor cells exhibiting partial-EMT traits showed upregulated VIM and ITGA5 expression, while maintaining near-normal epithelial marker expression.^110^ In ESCC, adhesion proteins such as VIM, CLDN1, and CLDN7 were used to classify tumor cells into epithelial, complete EMT, and partial-EMT subgroups.^111^ In p16-positive cervical cancer, partial-EMT cells demonstrated increased β-catenin expression with minimal changes in VIM and E-cadherin levels.^112^

Pastushenko et al. observed that partial-EMT subgroups displayed negative or low expression of CD106, CD61, and CD51, or expressed only CD106.^112^ During the early partial-EMT phase, epithelial markers such as E-cadherin and EpCAM were lost, but CK5, CK8, and CK14 were retained, disappearing only in the late EMT phase. Meanwhile, VIM and N-cadherin were highly upregulated in the early mixed state and remained elevated in the late partial-EMT phase. Late partial-EMT is strongly associated with increased expression of biomarkers such as cadherin-11, collagen XXIV transcription complex, FAP, MMP19, LOX-like 1 (LOXL1), and PDGFR α/β.^107^ Comprehensive scRNA-seq analysis of HNSCC tumors revealed several specific surface markers of partial-EMT, indicating TGFBR1, PDPN, LAMB3 and LAMC2, which were strongly associated with poor prognosis in independent patient cohorts.^113^ Additionally, high expression of lncRNAs, such as MYOSLID, in partial-EMT cells correlated with increased invasiveness and poorer prognosis, further implicating these lncRNAs in the progression of partial-EMT.^114^

A novel artificial intelligence (AI) model, trained on gene expression profiles from the NCI-60 cell line, has been developed to predict survival outcomes using clinical datasets.^115,116^ This model revealed that higher mixed EMT scores correlated with lower overall survival rates in lung cancer patients. However, this correlation did not extend to breast and ovarian cancers, suggesting that the prognostic significance of partial-EMT may vary by tissue type or that current models may not fully capture its complexity across different cancers.^115,116^ Overall, partial-EMT is characterized by distinct biomarkers, enhanced metastatic traits, and increased stemness. These features underscore its critical role in tumor progression and prognosis, highlighting the potential for targeting partial-EMT markers in clinical applications (Fig. 3).

## Targeting partial-EMT and combination immunotherapy

Therapies targeting partial-EMT primarily aim to inhibit the TGF-β pathway, which plays a dual role in tumor progression. TGF-β acts as a tumor suppressor in early cancer stages but shifts to a tumor-promoting role in advanced disease, driving partial-EMT, angiogenesis, and immune evasion.^117^ In the partial-EMT state, active TGF-β signaling particularly facilitates the transition of cancer cells to a mesenchymal phenotype, thereby increasing their invasiveness and metastatic potential.^16^ Two main classes of TGF-β inhibitor are under evaluation: small-molecule receptor kinase blockers, which synergize with PD-1/PD-L1 therapies, and antisense oligonucleotides, which reduce ligand production.^118^ Current research focuses on improving precision to minimize off-target effects.^119^ Clinical trials are in the early to mid-phase for solid tumors, with biomarker-guided, patient-specific approaches likely to achieve therapeutic benefits.

Immunotherapy, particularly immune checkpoint inhibitors, has shown significant efficacy in many solid tumors. However, monotherapy has limited success due to low response rates and substantial side effects.^120,121^ For instance, many OSCC patients exhibit limited responsiveness to immunotherapy, prompting research into combination strategies to improve efficacy while minimizing adverse effects.^122,123^ Combination therapies can enhance the cytotoxic functions of immune cells and inhibit tumor progression. In OSCC, pembrolizumab and nivolumab are FDA-approved immunotherapies.^124^ Clinical trials have shown that these therapies provide durable responses in some patients, particularly those with recurrent or refractory tumors. However, only a small fraction of OSCC patients benefit, leading to the exploration of combination treatments.^125^ For example, combining anti-angiogenic agents with PD-1 inhibitors has shown superior outcomes. A trial by Hua et al. demonstrated that combining anti-PD-1 inhibitors with anti-VEGF inhibitors achieved significant treatment responses in over 60% of patients.^126^ Bevacizumab, an anti-VEGF agent, not only inhibits angiogenesis but also enhances T cell proliferation and activity, significantly improving overall survival rates when combined with anti-PD-L1 inhibitors.^127,128^ EGFR inhibitors also play a critical role in OSCC by promoting tumor antigen presentation and enhancing CD8^+^ T cell activity.^129,130^ Cetuximab, a commonly used anti-EGFR monoclonal antibody, shows limited efficacy, with fewer than 20% of OSCC patients responding. However, combining cetuximab with anti-PD-L1 inhibitors improves median overall survival and quality of life in patients with high PD-L1 expression.^131^ TGF-β contributes to immune suppression by inducing the conversion of T cells into Tregs and MDSCs are upregulating PD-L1 and PD-1.^132–134^ Combining anti-TGF-β inhibitors with immune checkpoint inhibitors can significantly enhance treatment efficacy. For example, a study combining nivolumab with Galunisertib in cancer patients showed a higher overall survival rate compared to either therapy alone. This combination was well-tolerated, with minimal treatment-related adverse events.^135^

To better understand epithelial plasticity in OSCC and guide treatment selection, partial-EMT should be quantified based on its spatial ecology. A practical approach is to develop a margin-anchored partial-EMT score using multiplex IHC or spatial transcriptomics on annotated invasive fronts. This score integrates the co-expression of epithelial markers, alongside matrix-interaction and remodeling programs, as well as stromal features. This composite readout can be used to classify tumors into continuous value or stratified tiers, with thresholds established through discovery cohorts and validated externally.

Therapeutic stratification can follow from this framework. Tumors with a high partial-EMT score and limited CD8 infiltration are candidates for combination therapies coupling PD-1/PD-L1 blockade with TGF-β inhibition. Dosing and scheduling can be optimized to remodel the invasive margin while maintaining tolerability. High partial-EMT tumors exhibiting strong angiogenic or lymphangiogenic signals may benefit from adding anti-VEGF therapy to PD-1/PD-L1 blockade to normalize vasculature and relieve immune exclusion. When high partial-EMT coincides with elevated EGFR activity, combining EGFR inhibitors with PD-1/PD-L1 therapy can enhance antigen presentation and T-cell access. In tumors with matrix stiffening and FAK–YAP/TAZ signaling, inhibitors of matrix sensing or collagen cross-linking provide a rational partner for immunotherapy. Conversely, inflamed partial-EMT-low tumors with pre-existing effector-cell infiltration are more likely to respond to checkpoint monotherapy, with combinations reserved for secondary resistance or score escalation during treatment. Prognostically, higher partial-EMT scores correlate with infiltrative growth, nodal metastasis, and increased recurrence risk, and can be interpreted alongside PD-L1 expression, HPV status, and tumor mutational burden to refine risk stratification.

Implementation of this approach requires careful attention to reproducibility and dynamics. Sampling should target pathologist-annotated invasive fronts, controlling pre-analytic variables and tumor cellularity. Model training should use cross-validated discovery cohorts to determine marker weights and decision thresholds, followed by prospective validation. Pharmacodynamic monitoring can use paired biopsies at baseline and during treatment to assess whether combinations reduce the partial-EMT score and enhance effector-cell access at the margin. Liquid-biopsy surrogates, such as CTC clusters and exosomal transcripts, can provide complementary, low-burden readouts. Incorporating this score into trial design allows for prospective enrichment of patients most likely to benefit from TGF-β or matrix-directed combinations, supports early stopping rules based on on-treatment score reversal, and offers a concrete pathway from biological insight to clinical decision-making in OSCC.

Partial-EMT functions as a critical hub in OSCC metastasis and drug resistance, driving the evolution of a clinically aggressive phenotype. Combination therapies represent a promising strategy to enhance treatment efficacy and improve quality of life for these patients. Future research should prioritize integrating immunotherapy with targeted therapies against partial-EMT to achieve substantial improvements in survival outcome. Clinical trials targeting EMT and MET in cancer therapy are growing (Tables 1 and 2; https://clinicaltrials.gov/), highlighting the growing interest in modulating epithelial plasticity as a therapeutic approach. These trials provide valuable insights into how partial-EMT may be leveraged or inhibited in clinical settings, emphasizing the need for OSCC-specific strategies based on the unique molecular and immunological features of partial-EMT in this cancer type.

**Table 1:** Clinical trials related to targeting EMT in cancer treatment

NCT Number	Study Title	Study Status	Conditions	Interventions	Phases
NCT02602938	Aspirin on CTCs of Advanced Breast and Colorectal Cancer	UNKNOWN	EMT CTCs	DRUG: Aspirin	PHASE2
NCT03509779	Pronostic and Predictive Value of EMT in Localized Lung Cancer	RECRUITING	NSCLC, Stage I, II, IIIA, IIIB | Surgery Progression |EMT	NA	NA
NCT02022904	Prostate Cancer Circulating Tumor Cells Based on Epithelial-Mesenchymal Transition Biology	WITHDRAWN	Prostate Cancer	DEVICE: Near infrared (NIR) emissive nanotechnology	NA
NCT05550415	The Role of Simvastatin in the Epithelial-Mesenchymal Transition Process of Breast Cancer	RECRUITING	Triple Negative Breast Cancer | Chemotherapy Effect | Simvastatin Adverse Reaction	DRUG: Simvastatin 40 mg|DRUG: Placebo	PHASE2
NCT04137406	Role of SIRT1 in Regulation of Epithelial-to-mesenchymal Transition in Breast Cancer Lymph Nodes Metastasis	UNKNOWN	Breast Cancer | Lymph Node Metastases	NA	NA
NCT04323917	Detection of High Expression Levels of EMT-Transcription Factor mRNAs in Patients With Pancreatic Cancer and Their Diagnostic Potential	UNKNOWN	Pancreatic Cancer	DIAGNOSTIC_TEST: Liquid biopsy	NA
NCT04323813	“High Levels of EMT-TFs for the Diagnosis of Colorectal Cancer (CRC)”	UNKNOWN	Colorectal Cancer	DIAGNOSTIC_TEST: Liquid biopsy	NA
NCT01927354	Study on the Interplay Between Twist1 and Other EMT Regulators Through microRNA-29 Family.	UNKNOWN	HNSCC	NA	NA

*EMT* epithelial-mesenchymal transition, *CTC* circulating tumor cells, *NSCLC* non-small cell lung carcinoma, *HNSCC* head and neck squamous cell carcinoma

**Table 2:** Clinical trials related to targeting MET in cancer treatment

NCT Number	Study Title	Study Status	Conditions	Interventions	Phases
NCT04982224	Study of REGN5093-M114 (METxMET Antibody-Drug Conjugate) in Adult Patients With Mesenchymal Epithelial Transition Factor (MET) Overexpressing Advanced Cancer	RECRUITING	Advanced NSCLC	DRUG: REGN5093-M114|DRUG: Cemiplimab	PHASE2
NCT04077099	A Study of REGN5093 in Adult Patients With Mesenchymal Epithelial Transition Factor (MET)-Altered Advanced Non-Small Cell Lung Cancer	ACTIVE_NOT_RECRUITING	NSCLC	DRUG: REGN5093	PHASE2
NCT01697072	First-Line Treatment for Locally Advanced or Metastatic Mesenchymal Epithelial Transition Factor (MET) - Positive Gastric, Lower Esophageal, or Gastroesophageal Junction (GEJ) Adenocarcinoma	TERMINATED	Gastric Cancer	DRUG: Rilotumumab | OTHER: Placebo | DRUG: Epirubicin | DRUG: Cisplatin | DRUG: Capecitabine	PHASE3
NCT05541822	To Evaluate the Efficacy, Safety, Tolerability and Pharmacokinetic Profile of ABN401 in Patients With Advanced Solid Tumors Harboring c-MET Dysregulation	RECRUITING	Advanced Solid Tumors	DRUG: ABN401	PHASE2
NCT06669117	FIH Trial of VERT-002 in Patients with Locally Advanced or Metastatic Solid Tumors with MET Alterations	RECRUITING	Solid Tumor | MET Alteration	DRUG: VERT-002	PHASE2
NCT05110196	Study of Capmatinib in Indian Patients With MET Exon 14 Skipping Mutation Positive Advanced NSCLC	RECRUITING	NSCLC	DRUG: Capmatinib 150 mg | DRUG: Capmatinib 200 mg	PHASE4

*MET* Mesenchymal-Epithelial Transition, *NSCLC* Non-Small Cell Lung Carcinoma

## Current challenges and future perspectives

### Current challenges

**Fig. 4:**
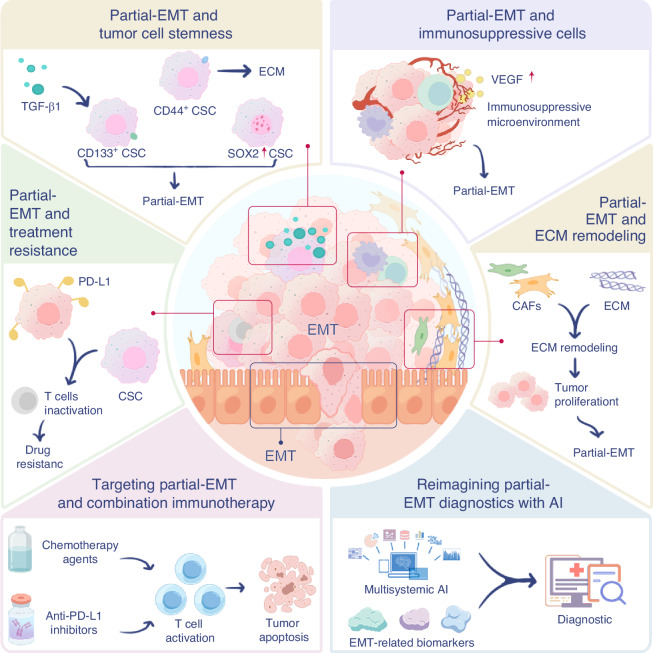
Multifaceted roles of partial-EMT in the tumor microenvironment and therapeutic strategies. Partial-EMT plays a central role in regulating the TME. It promotes tumor cell stemness (e.g., CD44⁺/CD133⁺/SOX2⁺ CSCs), induces immunosuppressive cell infiltration and VEGF-mediated angiogenesis, and drives ECM remodeling through CAFs, all contributing to tumor progression. Moreover, partial-EMT enhances treatment resistance by upregulating PD-L1 expression and facilitating immune evasion. Targeting partial EMT via combination therapies—such as chemotherapy and immune checkpoint blockade—can activate T cells and induce tumor apoptosis. In parallel, integrating multi-omics data and AI enables the identification of EMT-related biomarkers, offering new opportunities for precise cancer diagnostics and personalized therapy. This figure was hand-drawn by the author using Procreate (https://procreate.com)

Advancing research on the role of partial-EMT in OSCC presents numerous challenges. One major issue is tumor heterogeneity, as the behaviors of tumor cells during the EMT process vary widely across different patients, complicating the identification of reliable partial-EMT biomarkers. Additionally, partial-EMT is a dynamic, transitional process, influenced by factors such as the TME and the patient’s overall condition. Understanding the dynamic shifts in partial-EMT and uncovering the mechanisms driving cell state transitions remain significant hurdles (Fig. 4). Traditional experimental methods, including cell line cultures and tissue analyses, often fail to capture the full complexity of EMT, particularly with regard to cell-to-cell and cell-to-matrix interactions. Moreover, patient-derived tissue samples are prone to information loss during processing and analysis, especially when attempting to replicate active TME signaling pathways in vitro. While high-throughput sequencing technologies generate vast datasets, interpreting them effectively remains challenging due to their complexity and diversity. Current bioinformatics tools are often inadequate for meaningful analysis specific to partial-EMT. Furthermore, the lack of standardized methodologies and assessment criteria across studies hampers the comparability of findings and slows overall research progress. Overcoming these challenges will require innovative approaches, collaborative efforts, and the development of new tools and frameworks to deepen our understanding of partial-EMT in OSCC.

In OSCC, combining anti–TGF-β agents with immunotherapy has yielded limited and inconsistent benefit. This is partly due to intratumoral and stromal heterogeneity, leading to variable TGF-β dependence and spatially confined partial-EMT and immune-exclusion programs, typically localized at the invasive front—an area often missed in routine biopsies. Additionally, tumors rapidly activate compensatory pathways such as IL6/STAT3, AXL/YAP and WNT signaling, undermining therapeutic efficacy. The tumor microarchitecture is typically mosaic, with partial-EMT cells at the leading edge interspersed with epithelial clusters and CAF-rich stroma. As a result, single-site biopsies and bulk assays may fail to detect spatially restricted states, causing biomarker misclassification and diluted treatment responses. Even in tumors with high TGF-β activity, blockade may be bypassed through stromal or tumor-intrinsic mechanisms maintaining motility and immune evasion.^136^ Clinical constraints, such as dose-limiting toxicities and prior therapy-induced stromal alterations, further limit response and may explain the frequent observation of disease stabilization without durable remission in unselected OSCC cohorts.

Clinically translating partial-EMT requires overcoming challenges in analytical validity, clinical relevance, and reproducibility. The common use of VIM and E-cadherin co-expression is limited by pre-analytical variability, antibody inconsistencies, and difficulty distinguishing membranous from cytoplasmic E-cadherin. Moreover, as partial-EMT is concentrated at the invasive margin, random sampling often underestimates its presence. Biological plasticity complicates interpretation, as therapies can transiently suppress E-cadherin without inducing a full EMT program. Robustness varies by OSCC subtype—keratinizing tongue and gingival tumors with dense CAF infiltration often show stronger partial-EMT and TGF-β activity than well-differentiated or HPV-positive tumors.^137^ To improve reproducibility, composite and spatially informed assays are recommended over single marker pairs. Practical options include multiplex IHC panels combining epithelial and mesenchymal/matrix markers at the invasive front.^138^ Spatial transcriptomics or microdissected-margin RNA profiling can complement these protein-based assays. Prospective studies should predefine cutoffs, target the invasive front, and stratify by anatomic site, differentiation grade, and HPV status.

Therapeutically targeting partial-EMT is biologically and ethically complex. Since EMT and MET are reversible and context-dependent, suppressing EMT may inhibit dissemination but could also promote MET-driven metastatic outgrowth if cells have already seeded distant niches.^139^ Moreover, targeting TGF-β and matrix-remodeling pathways may disrupt mucosal repair, immune balance, and tissue integrity. These uncertainties raise ethical concerns about unintended consequences, especially in patients at risk for latent metastasis.

Technical challenges further complicate clinical application. As partial-EMT is spatially and temporally dynamic, single biopsies and bulk readouts often miss key regions. Assay accuracy depends on detecting membranous E-cadherin and co-expressed mesenchymal and matrix markers, yet variability in platforms and sampling introduces inconsistency. Given the plasticity of cell state and rapid compensatory feedback via IL6–STAT3, AXL/YAP, and WNT circuits, pathway-specific inhibition may not result in lasting phenotypic changes.^140^ Therefore, robust trial designs should employ spatially tuned composite biomarkers, standardized sampling at the invasive margin, and harmonized thresholds across centers.

An ethically sound clinical approach should focus on reversible, well-monitored interventions. Window-of-opportunity trials in resectable OSCC could track cell-state transitions at the invasive margin using multiplex pathology, spatial transcriptomics, and liquid biopsies (e.g., circulating tumor DNA [ctDNA], CTCs), while monitoring for unintended MET activation at distant sites. Adaptive trial designs with stopping rules for accelerated colonization, independent safety monitoring, and transparent informed consent are essential. In parallel, equity and privacy concerns must be addressed as spatial and AI-enabled diagnostics are introduced. Algorithms must be audited for site or scanner bias, data privacy must be protected, and access to testing should be equitable. Ultimately, addressing these ethical, technical, and biological constraints will determine whether partial-EMT becomes a clinically actionable target in OSCC—or remains a promising but uncertain concept.

### Future perspectives

Addressing the challenges posed by partial-EMT in OSCC will require coordinated efforts across several key areas. First, the integration of multi-omics data—including genomics, transcriptomics, proteomics, and epigenomics—will be essential for elucidating the molecular mechanisms and regulatory networks that drive partial-EMT. This systems-level approach can identify key regulators and signaling pathways, clarifying the complex relationship between EMT states and tumor progression. Advanced sequencing technologies, particularly single-cell and spatial platforms, enable detailed profiling of cellular phenotypes and interactions within the TME. These tools allow researchers to assess the stability of hybrid states and map their spatial context, offering insight into their biological and clinical relevance. Emerging spatial technologies, such as spatial transcriptomics and proteomics, offer practical solutions to current limitations in studying partial-EMT. By directly mapping gene and protein expression within tissue architecture, these tools can reveal the enrichment of partial-EMT programs at the invasive front and quantify their spatial associations with CAFs and immune cells. Spatial readouts can also distinguish early and late partial-EMT states by detecting coordinated expression of motility, matrix-interaction, and immune-exclusion modules—while preserving epithelial features. Targeted spatial panels focusing on epithelial, mesenchymal, matrix-remodeling, and TGF-β response genes can be applied to routine core biopsies, enabling harmonized scoring across centers and time points. This approach mitigates under-sampling, guides microdissection, and enables reproducible, region-specific assessments. In parallel, mathematical modeling and simulation of EMT-related signaling networks can help predict therapeutic outcomes and optimize intervention strategies. By modeling feedback loops and compensatory circuits, these methods can inform drug design and combination strategies targeting partial-EMT. AI, particularly deep learning, offers powerful tools for analyzing complex datasets. AI can enhance the interpretation of spatial data and routine pathology. Weakly supervised models applied to whole-slide HE images can infer hybrid epithelial–mesenchymal morphology and estimate the fraction and distribution of partial-EMT at the invasive margin. Graph-based learning from multiplex imaging data can integrate cell identity, spatial relationships, and stromal context to detect collective migration and immune-exclusion patterns. AI-derived uncertainty maps can identify regions in small biopsies that require re-sampling, while domain adaptation methods ensure robust performance across different scanners, stains, and anatomic sites. On the molecular side, AI-enabled deconvolution techniques can infer partial-EMT signatures from bulk RNA-seq data, aligning them with spatial or IHC results for cross-platform validation. These spatial and computational tools support the development of a composite, biopsy-deployable partial-EMT score that integrates epithelial-mesenchymal intensity at the invasive front with stromal and immune-exclusion features. Such a score can be used to present patients for anti-TGF-β combination trials, monitor pharmacodynamic responses and benchmark performance across OSCC subtypes in terms of analytical validity, clinical validity, and clinical utility.

Ultimately, rigorous, biomarker-driven translation of these insights into clinical practice will be crucial for improving patient outcomes. Developing targeted therapies for partial-EMT, combined with personalized treatment strategies, can enhance therapeutic efficacy and quality of life. Carefully designed clinical protocols—such as window-of-opportunity trials, adaptive designs, and real-time biomarker monitoring—will help bridge the gap between molecular research and clinical application. By pursuing these directions, the field can move closer to overcoming treatment resistance in OSCC and realizing the promise of precision oncology.

## Conclusion

In the partial-EMT state, cancer cells adopt a hybrid epithelial–mesenchymal phenotype that increases invasive capabilities. This plastic state enables tumor cells to dynamically interact with the TME, facilitating their adaptation to changing environmental cues. Through interactions with immune and stromal cells, partial-EMT reshapes the TME into an ecosystem that promotes cancer progression (Fig. 4). Additionally, partial-EMT cells often acquire stem cell-like characteristics, contributing to their high plasticity and heterogeneity. These traits render them highly resistant to therapies such as chemotherapy, posing significant challenges for effective treatment. Understanding the molecular mechanisms and signaling pathways driving partial-EMT is crucial for developing innovative cancer therapies. Targeting partial-EMT in future therapeutic strategies holds the potential to improve treatment outcomes, ultimately enhancing both survival rates and the quality of life for cancer patients.
